# Humidity-Sensing Mattress for Long-Term Bedridden Patients with Incontinence-Associated Dermatitis

**DOI:** 10.3390/mi14061178

**Published:** 2023-05-31

**Authors:** Jinpitcha Mamom, Phadungsak Ratanadecho, Chatchai Mingmalairak, Bunyong Rungroungdouyboon

**Affiliations:** 1 Department of Adult Nursing and the Aged, Faculty of Nursing, Thammasat University, Pathum Thani 12121, Thailand; 2 Center of Excellence in Electromagnetic Energy Utilization in Engineering, Department of Mechanical Engineering, Faculty of Engineering, Thammasat University, Pathum Thani 12120, Thailand; ratphadu@engr.tu.ac.th; 3 Department of Surgery, Faculty of Medicine, Thammasat University, Pathum Thani 12120, Thailand; michatch@staff.tu.ac.th; 4 Center of Excellence in Creative Engineering Design and Development, Faculty of Engineering, Thammasat University, Pathum Thani 12121, Thailand; rbunyong@engr.tu.ac.th

**Keywords:** humidity sensors, mattress design, incontinence-associated dermatitis, bedridden patients

## Abstract

Designing new medical devices with advanced humidity sensors is of great significance for patients with incontinence-associated dermatitis (IAD). The primary goal of this study is to test the humidity-sensing mattress system for patients with IAD in clinical settings. The design of the mattress is set at 203 cm, with 10 × 3 sensors, dimensions of 19 × 32 cm, and a weighted bearing of 200 kg. The main sensors consist of a humidity-sensing film, a thin-film electrode (6 × 0.1 mm), and a glass substrate (500 nm). The sensitivity of the test mattress system showed that the resistance-humidity sensor was at a temperature of 35 °C ($V_{0\;}$ = 30 V, $V_{0\;}$= 350 mV), with slope at 1.13 V/fF, f = 1 MHz, 20–90% RH, and a response time of 20 s at 2 μm. In addition, the humidity sensor reached 90% RH, with a response time of less than 10 s, a magnitude of 1$0^{7}$–1$0^{4}$ Ω, 1 mol%, ${\mathrm{CrO}}_{1.5}$, and F$O_{1.5}$, respectively. This design is not only a simple, low-cost medical sensing device, but also opens a new pathway for developing humidity-sensing mattresses in the field of flexible sensors, wearable medical diagnostic devices, and health detection.

## 1. Introduction

Medical mattresses with advanced humidity sensors are receiving significant attention when it comes to treating patients with IAD [1,2,3]. Designing the humidity transducer so that it can detect an amount of water vapour ($H_{2}$O) is one of the most important medical judgements and diagnoses. The recent development of humidity sensors for patients with IAD is gaining acceptance within medical devices [4,5,6]. Some scholars have developed a humidity sensor with inflammatory cytokines [7], skin temperature [8], metal oxide nanomaterials [9], transepidermal water [10], and water contact angle [11]. In the present designs, there has been a testing of mattresses which sense relative humidity (RH) in terms of resistance [5,12,13,14], film thickness [15], and the refractive index (RI) [16].

Humidity sensors for patients with IAD have been tested with moisture-associated dermatitis, liquid, and water vapour [1,8,10]. The humidity-sensors system has acquired water vapour consisting of highly reactive dipolar molecules, temperature, gaseous form, moisture, and liquid [1,6,9]. Previous studies have tested the humidity sensors’ design based on sensitivity, electro-active flow control devices, fast response, and low cost for sensing materials [4,17]. Many scholars have divided patients into three types when it comes to IAD-sensing devices: light, moderate, and heavy skin damage [18,19,20]. Humidity sensors are mainly based on impedance and resistance, including metal oxides [21], perovskites [22], and organic polymers [23].

Typically, the traditional design of humidity-sensing mattresses for patients with IAD has limitations, such as early IAD diagnosis [2,7]. Indeed, patients with IAD range from 5.3–46.1% in residential care and 19–54.7% for critically ill patients [24]. Previous scholars have tested the design of humidity sensors according to the clinical setting at 6% of moisture-associated skin damage in residential care [25]. Matar et al. [8] provided four types of sensing moisture-associated skin damage in IAD: erythema, localised swelling, vesicles, and crusting/scaling. The humidity sensor for IAD patients includes keeping the skin dry, maintaining pH balance, and offering moisturiser [26]. Despite this, mattresses using a flexible humidity sensor constitute one of the most essential medical devices, with examples being polymer film-based sensors [2], paper-based sensors [27], and flexible fibre-optic humidity sensors [28].

Thus, there still remains a lack of advanced humidity sensors which have been tested with patients exhibiting early signs of IAD. Indeed, the characteristics of sensing moisture-associated skin damage are limited, focusing on different types of sensing materials [1]. Previous studies have set dimensions of 10 × 5 cm to cut a point on the sensitive fibre-optic mattress [29]. A humidity sensor for IAD patients has the potential to increase the quality of devices, comfort, and sensitivity. The design includes using a thin firm electrode and glass substrate, which greatly tests the sensitivity of the humidity-sensing mattress for patients with IAD. Hence, the thin film electrode as an adhesion agent is reliable for mattress design but has not yet been applied to humidity sensors.

Accordingly, our study has designed a humidity-sensing mattress for patients with IAD following exposure to experimental testing in clinical settings. Firstly, the design of the mattress consists of a humidity-sensing film, a thin film electrode, and a glass substrate. Secondly, the design is also focused on the stability of the humidity sensors (RH, AB, and D/F PT), ambient temperature, and adsorption–desorption dynamic cycles. Finally, when testing the response time of 10–120 s with five volts, the magnitude ranged from 1$0^{7}$–1$0^{4}$ Ω, and the pore ranged from 0–10 μm. The results of this study attest not only to humidity sensor designs for IAD patients but also the development of sensitive mattresses which can be developed in clinical settings.

## 2. Methods

### 2.1. Humidity-Sensing Design

**Figure 1:**
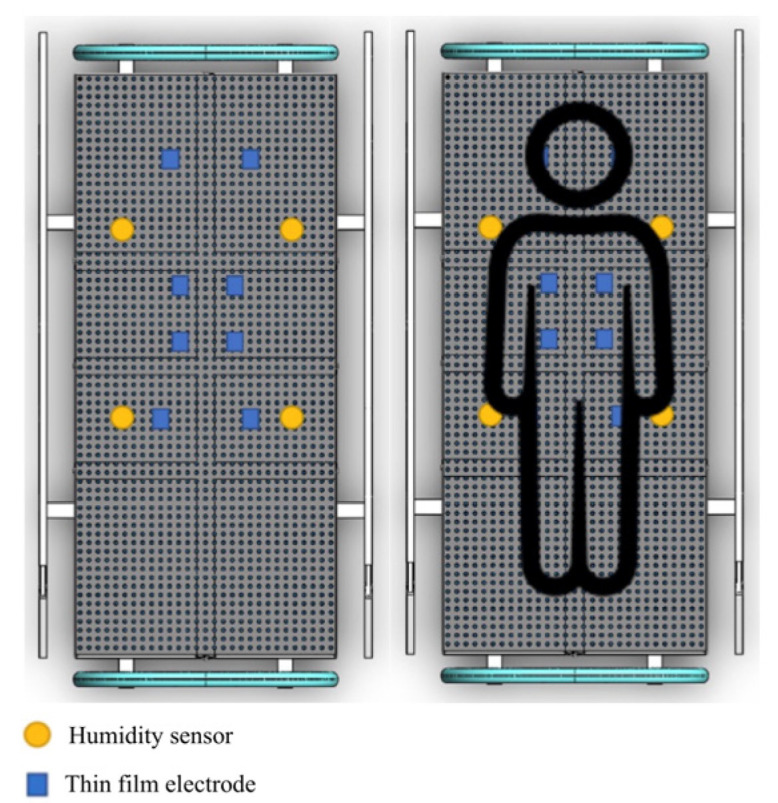
Humidity-sensing mattress design.

When it comes to the sensing design of the mattress environmental chamber (MEC), it tests individual weight-loading support of 200 kg. The MEC consists of a lengthwise, zipped opening to allow mattress placement, as well as a loading array. The design of the testing mattress has a set of 203 cm × 96.5 cm peripheral outlets and a moisture-bearing patient moisture reservoir (PMR). The PMR rests on humidity sensors consisting of 0.28${\;m}^{2}$ (0.46 m × 0.6 m) polypropylene and a nominal total wet mass of 195 g (44 g dry). The water bladder measures approximately 0.43 $m^{2}$ (0.46 m × 0.91 m) and completely overlays the PMR. Figure 1 presents the humidity-sensing mattress design. The sensor region consists of two position areas as follows:

- The humidity sensor is real-time monitoring of wetting moisture, water vapour, and vibration urine for converting the data into a corresponding electrical signal;
- The thin-film electrode array is a method for the fabrication of submicrometer gold on polyester and silicon. The sensor structure consists of a pair of interdigital electrodes and aluminium films on the glass substrate for the thickness (500 nm).

### 2.2. Humidity-Sensing Principle

A humidity sensor tests the amount of water vapour and temperature [1]. It is worth noting that a sensitive term encompasses (i) relative humidity (RH), absolute humidity (*AH*), parts per million (PPM), and the dew/frost point (D/F PT). The humidity sensor classifies the mass of water vapour, in grams per cubic metre, and feet (1 grain = 17000  pound (lb), as in the following equation:

(1) $$\mathrm{AH}=\frac{M_{w}}{v}$$

where *AH* is the g/$m^{2}$ or grain/$ft^{2}$, $m_{w}$ is the mass of water vapour, and v is the volume of air (m^3^ or *ft*^3^). The RH is tested as the ratio of moisture to the maximum level, which gives the temperature and humidity. In the following, RH is equated as:

(2) $$\mathrm{RH}\%=\frac{P_{V}}{P_{S}}\;\times \;100$$

where $P_{V}$. is the actual partial moisture and $P_{S}$ is the saturated temperature. It is known that saturation humidity (*SH*) is defined as:

(3) $$\mathrm{SH}=\frac{M_{ws}}{v}$$

where *SH* is the g/$m^{3}$, $m_{ws}$ is the mass of water vapour (g), and v is the volume of air ($m^{3}$). *SH* is the temperature in a unit volume of gas. *RH* calculates the ratio of *AH* to *SH* as follows:

(4) $$\mathrm{RH}=\frac{AH}{SH}\;\times \;100$$

The PPMv is defined as the volume of water vapour in dry gas, the PPMw refers to *the* water *vapour*. The PPMv and PPMw are amongst the A*H*, the D/F is based on the temperature (above 0 °C), and PT is the temperature (below 0 °C). However, the D/F point parameters are the A*H*.

The sensitivity of humidity is determined with the fixed capacity *of voltage* ($C_{0}^{\prime }$ = $C_{0}$ + $C^{\prime }$), as follows:

(5) $$V_{o}=V+V_{t}\;\frac{\Delta C}{2C_{0}+2C\prime +\Delta C}$$

The sensing sensitivity (S) is defined using the following equation:

(6) $$S=\frac{V_{97\%}-V_{11\%}}{97\%\;\mathrm{RH}-11\%\;\mathrm{RH}}\;\times \;100\%$$

where $V_{97\%}$ and $V_{11\%}$ represent the voltage of RH = 97% and RH = 11%. The S of humidity is approximately 29.9 mV/%RH.

Shimizu et al. [30] tested the field-effect transistor (FET) on host-PET devices and n-channel MISFET. The FET was set upper and lower on electrical connection with large resistance $R_{B}$, whilst the outcome voltage, $V_{out}$, was related to the capacity membrane, $C_{s}$, as follows:

(7) $$V_{out}=V_{0}R_{L}g_{m}\;(1+C_{1}/C_{s})$$

where $R_{L}$ is the load resistor to the electrode, $g_{m}$ is the transconductance of the PET, and $C_{1}$ is the voltage capacity of the gate insulator. Whilst $C_{2}$ is dependent on RH, $V_{out}$ is correlated with RH, the hysteresis is less than 3% RH, and there is a response time of 30 s. The capillary condensation of water vapour is cylindrical up to $r_{K}$, as follows:

(8) $$r_{K}=\frac{2\gamma M}{\rho RT\;\mathrm{In}\;P_{s}/P}$$

where $r_{K}$ is rious, γ, ρ, and M represent the surface tension, density, and molecular weight of water, and $P_{s}/P$ represents vapour. Shimizu et al. [30] defined the simulation analysis in two actual porous ceramic elements, MgA$I_{2}O_{4}$ and MgF$e_{2}O_{4}$. These elements are large parasites with a wide pore size in humidity using MgC$r_{2}O_{4}-T_{1}O_{2}$ for multifunctional sensors, which ranges from 20–90% RH, up to 150 °C, and with a response time of 10–120 s. The sensing elements are a tiny, porous, rectangular body of water containing MgC$r_{2}O_{4}-T_{1}O_{2}$ spinel solid solutions.

A porous $T_{1}O_{2}-V_{2}O_{5}$ sensor is based on additive and effective control (1 mol%), electrical conductivity, the pore size distribution, and response time (10 s). A pure MgF$e_{2}O_{4}\;$ element is detected, with the addition of 2 mol % of $K^{+}$, $L_{1}$ or $Na^{+}$ over the whole range of RH. In response to the temperature, a semiconductor uses $Sr_{1-x}La_{x}SnO_{3}$ and $SrO_{2-}MgO$. The electronic-type sensor was operated with d c voltage interferences of the elements with Pt/A$1_{2}O_{3}$.

### 2.3. Humidity-Sensing System

**Figure 2:**
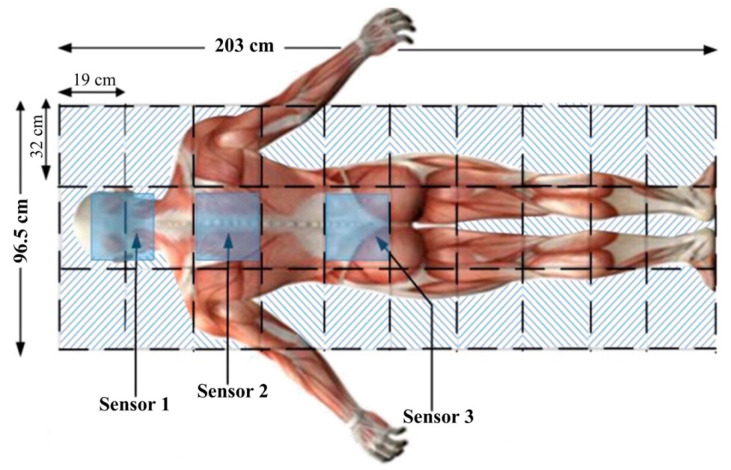
Patient body sensing position.

**Figure 3:**
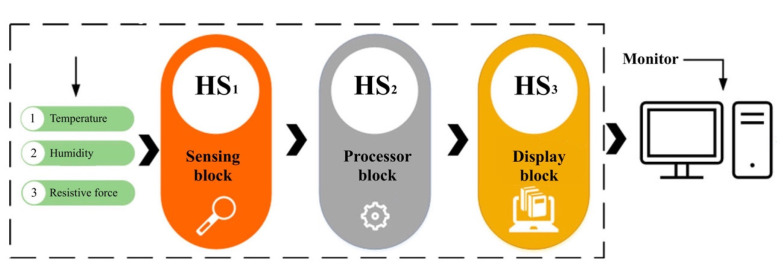
Humidity-sensing system.

The humidity-sensing mattress is designed for clinical settings: 203 × 96.5 cm, 10 × 3 sensors, and a dimension of 19 × 32 cm [31]. The physical human, the size, material devices, and contact points are considered in the clinical design. The body anatomy includes the head, neck (occipital muscle), shoulders (right and left shoulders, main rhombic muscles), and lumbar spine. The main system consists of three regions: sensing block, processor block, and display block. The body-sensing positions are depicted in Figure 2. The humidity-sensing system is shown in Figure 3.

### 2.4. Humidity-Sensing Function

**Figure 4:**
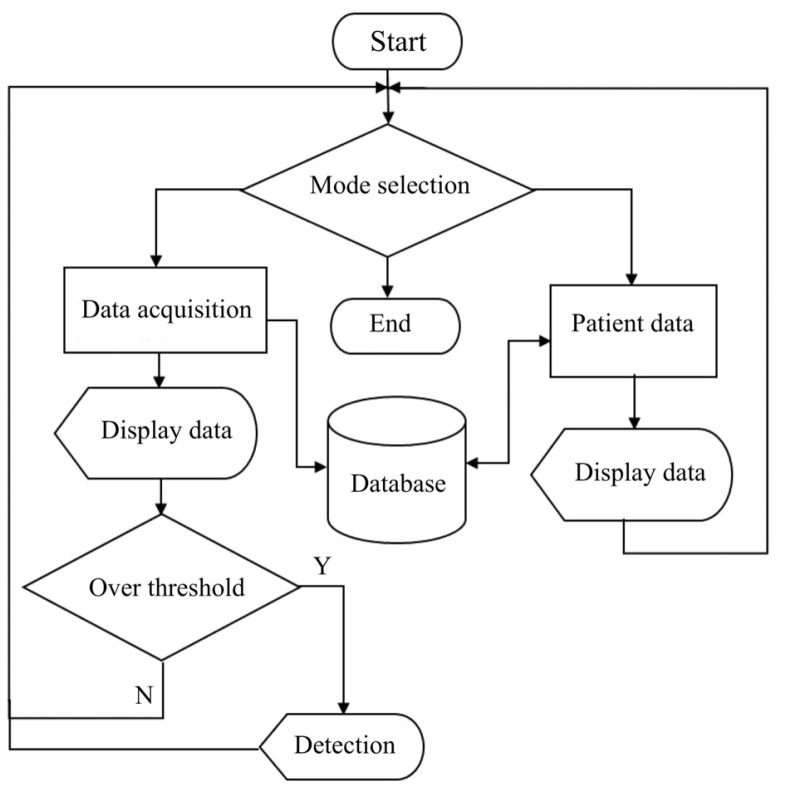
Flowchart of humidity-sensing system.

**Figure 5:**
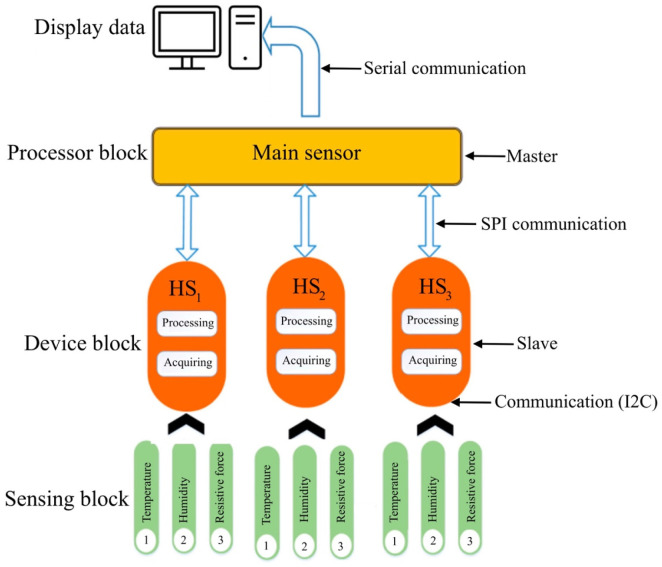
Data flow of humidity-sensing block system processing diagram.

**Figure 6:**
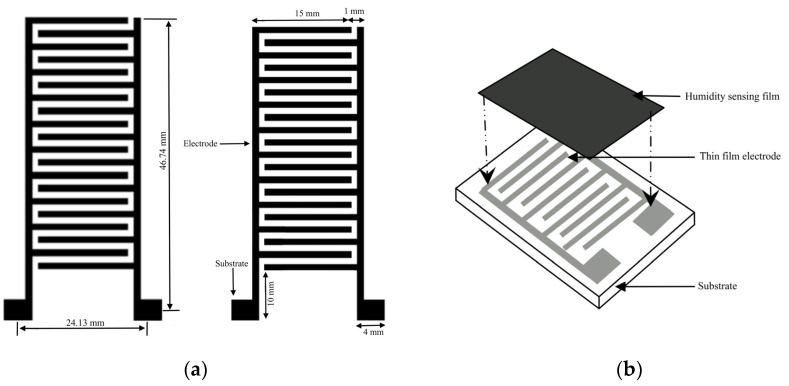
(**a**) Humidity-sensing film; (**b**) Schematic drawing of the humidity sensor.

In the humidity-sensing mattress, the recording functions include system setup, data storage, graphing, and an alarm. Sensitivity is performed using a system calibration with preferences regarding the humidity sensor. Data storage is recorded as historical records and current data. The graphing function allows the patients and their caregivers to observe the recording. The sensor will present a signal on the screen if a hazard over the threshold is detected. The flowchart of the humidity-sensing system is shown in Figure 4, whilst the humidity-sensing diagram is illustrated in Figure 5. The electrode film and schematic drawing of the humidity sensor can be seen in Figure 6a,b.

### 2.5. Humidity-Sensing System Platform

**Figure 7:**
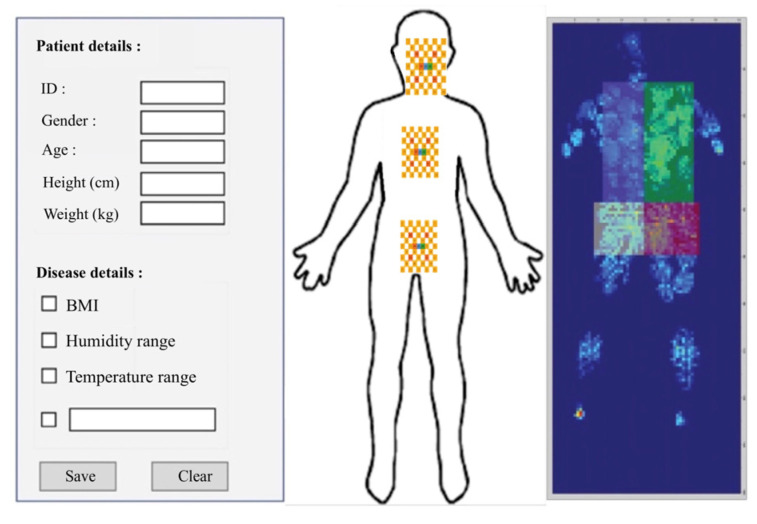
Humidity-sensing system platform.

In the warm-up system, with regard to the humidity-sensing mattress platform, there is a connection between ScreenView and the processor (serial communication, temperature, and humidity value). The response time is set at 1 min at 100 Hz on Arduino technical data. Analogue inputs of the Arduino board are used to extract data at FSR400. The mapping of the contact point of the humidity-sensing platform is shown in Figure 7. Table 1 presents the working humidity-sensing system platform.

### 2.6. Humidity-Sensing Volunteers

The testing of long-term bedridden patients with IAD is arranged with 19 volunteers of different temperature and humidity datasets. All volunteers are tested from the same number of patients with different values of BMI and pH index (see Table 2). To ensure testing accuracy, each volunteer is tested 10 times for 30 min in different body positions: lying, like side, supine, prone, and so on. In addition, the physical position of lying covers all three main areas that play a critical role in mattress contact. In this design, there are three effective contact points (ECP): occipital muscle, rhomboid major muscles, and spine endpoint. The testing sensor is initially put into standby mode for 1 min prior to each test, with ambient °C and RH being recorded. The humidity values have an arranged difference between °C and RH loading on the sensor datasets.

## 3. Results

### 3.1. Experimental Testing

A total of 19 voluntary patients with IAD underwent experimental testing at the Thammasat University Hospital, Thammasat University Rangsit Campus, Pathum Thani, Thailand. All patients provided informed consent to participate in the study. The Human Research Ethics Committee of Thammasat University (Science), Thailand, approved the study project in accordance with compliance with the Declaration of Helsinki, the Belmont Report, CIOMS guidelines, and international practice (ICH-GCP) (COA 032/2566 Project No. 66NU016). The body position regions consist of three sensors in patients with IAD: sensor 1 is the head and neck (occipital muscle), sensor 2 is the shoulders (rhomboid major muscles), and sensor 3 is the lumbar spine (spine endpoint), as can be seen in Figure 2 and Figure 7. The system starts by placing the 2.5 cm of thin film electrode without physical pressure (age, weight, and height) and temperature data are recorded as the initial value.

We designed the mattress so that it consists of a humidity sensor on the surface of the substrate and electrodes. A thin-film electrode is built on the glass substrate, which detects thickness at 500 mm. The length and width of the thin-film electrodes are approximately 0.1 mm, with two substracts (10 × 4 mm), as can be seen in Figure 6. The thickness of the layers is 15 μm in vacuum circumstances at 380 °C for 1 h. The humidity sensor includes the 8-bit microcontroller for temperature outputting and humidity values as serial data. Table 3 depicts the RH of different saturated solutions, whilst Table 4 illustrates the average of HR changes for FSR and the force sensor. Table 5 presents the values of the physical force, temperature, and humidity sensors.

The experimental data show that SHT10, DS18B20, and FSR 400 are inputs, based on the mean values of the recorded data. The aqueous solutions of humidity sensors for long-term bedridden patients with IAD are set as LiCI, MgC$I_{2}$, $K_{2}{\mathrm{CO}}_{2}$, Mg(N$O_{2}{)}_{2}$, NaCI, KCI, and $K_{2}$S$O_{2}$. The RH yields atmosphere values of 20–90%, which can be used to carry out an automatic RCL metre-PM. The testing frequency of humidity sensors varies from 50 Hz to 1 MHz in FSR sensors. The basis for the threshold comparison is an assumed mean water vapour mixing ratio of RH and sensor data. The threshold RH and humidity sensing assessment are shown in Table 6.

### 3.2. Voltage Sensitivity of RH Testing

**Figure 8:**
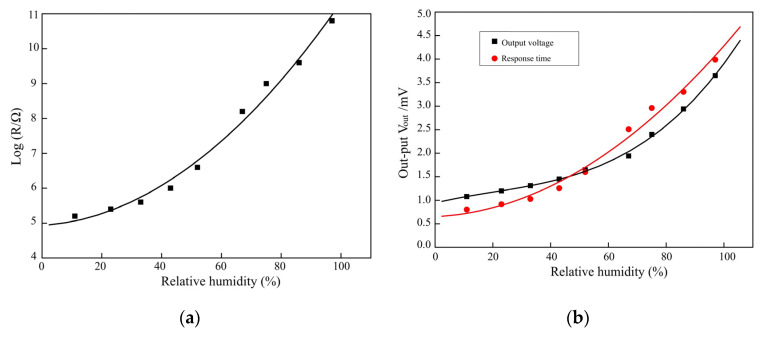
(**a**) RH sensor testing; (**b**) Voltage outcome of sensors for RH testing.

This principal part of the sensor is nodulised under a vacuum ranging from 30–100 °C for 30 min, with the voltage signal of 0.85 V for the same RH change. The sensor resistance is extracted from 10 cycles, and the humidity is changed to 5% HR with a 10 s change ratio at a constant temperature of 35 °C. The resistance-humidity characteristics of the sensor are 35 °C, voltage ($V_{0\;}$= 30 V, $V_{0\;}$= 350 mV), RH from 10% to 80%, and response and recovery times of <10 s and <5 s, respectively. The humidity sensor is completely returned to the initial value when compared with recovery time and response time. This is due to the different RH dielectric constant water vapour threshold (>7.30), liquid (close to 80), and slope (1.13 V/fF), whilst changing from 50 to 70% HR. The voltage and power consumption range between 5 V and 6.5 mW, with RH ranging from 20–90% with 3.50 V from 1.03 V. Testing of the RH and voltage outcome is carried out as seen in Figure 8a,b.

### 3.3. Humidity-Sensing Stability Testing

The humidity-sensing conductance at different response times and recovery times is obtained from the HR. The conductance is set at f = 100 Hz (0.10%), which tests 10 times at f = 1 MHz. The repeatability is obtained from adsorption–desorption dynamic cycles (f = 1 MHz) with 20–90% RH. The humidity sensor shows that different parameters of RH are increasing and decreasing (ranging from 10%–70%). The response time (humidification ranges from 25–85.5%) is approximately 20 s, and the recovery time (desiccation ranges from 25–85.5%) is approximately 10 s. In the RH ranges from 20% to 90%, the data can be well fitted using the formula log C = 0.015 RH + 0.099 under the frequency of f = 1 MHz. The sensor shows a maximum humidity hysteresis of 1.99%, corresponding to 90% RH. Table 7 depicts the characteristics of humidity-sensing repeatability.

**Figure 9:**
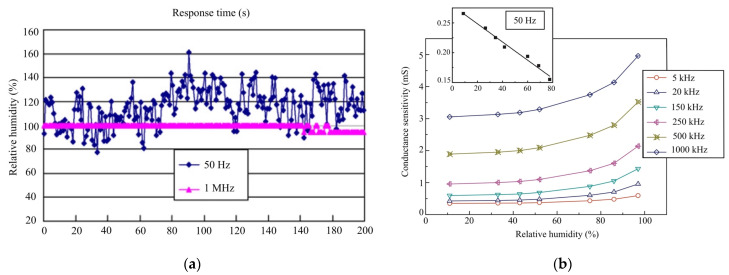
(**a**) Stability of humidity-sensor testing; (**b**) Conductance sensitivity of RH testing.

The resistance humidity sensors show water vapour molecules of hydroxyl ions on the surface of the C$r^{3+}$ sites. The porosity of MgC$r_{2}O_{4}-T_{1}O_{2}$ ranges from 30–40%, increasing by 1–2 μm and 300 mm. The operated sensor is set at 35 °C as the formal desorption. The sensitivity shows that RH ranges from ~15–90% at 100 Hz. The humidity-sensing properties of magnesium ferrite (MgF$e_{2}O_{4}$) are based on ratios of lithium (M$g_{1-x}{\mathrm{Li}}_{x}$F$e_{2}O_{4}$) at 0.2 ≤ x ≤ 0.6. The repeated testing of 10 cycles shows good reversibility of humidity sensor switching between 20% and 90% RH with 20 s. The conductance variation from RH 20%–RH 95.5% at f = 1 MHz is set at 20 cycles at 50 Hz; the stability sensor improves as the testing frequency increases. Figure 9 presents the stability of humidity sensors and conductance sensitivity.

### 3.4. Humidity-Sensing Performance Testing

The humidity-sensing performance testing is set at 35 °C, with a range of 20–90% RH. Three main humidity-sensor areas of patients with IAD (occipital muscle, rhomboid major muscles, and spine endpoint) are repeatedly tested with calibration changes at 0.5% RH, voltage ($V_{0\;}$= 30 V, $V_{0\;}$= 350 mV), f = 1 MHz, and response time (20 s). The response time constant of the HR is 10 s with a five-volt voltage regulator and a DIN plug connector. The design of the testing humidity sensor produces an accuracy of ±2% RH at 35 °C, with ±0.8% of the threshold water vapour, and ±1% stability. There is a small RH effect of −0.22% RH/°C at 100 °C, decreasing to −0.07% RH/°C at 0% RH. The characteristics of humidity-sensing repeatability testing are depicted in Table 8.

**Figure 10:**
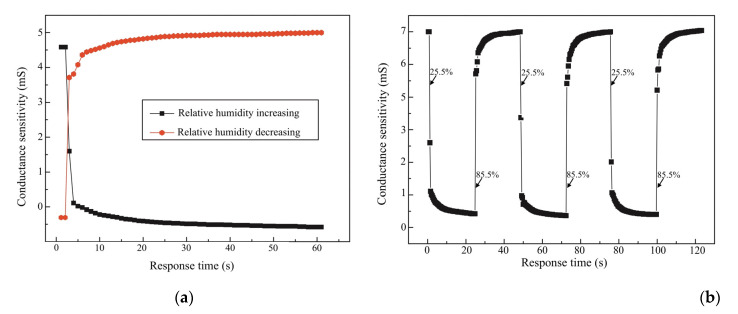
(**a**) Response and recovery curve of humidity-sensor testing; (**b**) HR and conductance of f= 1 MHz.

The characteristics of the experimental testing range from 20–90% RH at 35 °C, producing faster response and recovery times of between 20 s and 60 s. The thin-film electrode sensors are set as ${\mathrm{ZnCr}}_{2}O_{4}-{\mathrm{TiO}}_{2}$ and MgC$r_{2}O_{4}-T_{1}O_{2}$, showing a good linear response to 90% RH with 10 s. The RH ranges from 25–85%, when combined with a sensitivity of 0.43%/HR% at 30 °C of 10 s. The resistance changes at a magnitude of 1$0^{7}$ to 1$0^{4}$ Ω. The thick film indicated that nano-zirconium oxide (${\mathrm{ZrO}}_{2}$), a silicon substrate, a grain size of 20 nm, with a range of 25–85% RH, and a range of 50 Hz to 100 kHz. The testing of the humidity-sensing mattress illustrates a sensitivity of 1 mol% ${\mathrm{CrO}}_{1.5}$ and F$O_{1.5}$ to ${\mathrm{ZrO}}_{2}-{\mathrm{TiO}}_{2}$, ranging from 25–95% RH with a pore size of 0.1 μm and a response time of 10 s. Figure 10 illustrates the RH measured in sensing mattress performance.

## 4. Discussion

This is the first study to develop a new sensing mattress for patients with IAD to test the humidity-sensor designs. The design is expected to serve as a sensing device in clinical settings, providing an objective database through which to validate humidity-sensor designs. The volunteer tests showed resistance-sensing humidity at 35 °C, a slope of 1.13 V/fF, and f = 1 MHz with 20–90% RH. The sensors showed a response time of 20 s at 2 μm, and 300 mm. Our repeatable sensitivity increased to −0.22% RH/°C at 100 °C, decreasing to −0.07% RH/°C at 0% RH. The characterisation of the humidity sensor designs showed a short response of 10 s, magnitude at 1$0^{7}$–1$0^{4}$ Ω, 1 mol%, and ${\mathrm{CrO}}_{1.5}$, F$O_{1.5}$, respectively. We found that resistance values of the different parameters were close to 37 °C (i.e., ±1 °C), with values in the range of 20–80% RH, and air velocity of 0.06–0.3 m/s.

The stability of the humidity values is also an important factor affecting the sensitivity of three main sensors: the occipital muscle, rhomboid major muscles, and spine endpoint (see Table 4 and Table 5). The highest frequency changes up to f = 1 MHz were observed for three sensors of magnesium ferrite (MgF$e_{2}O_{4}$) on ratios of lithium (M$g_{1-x}{\mathrm{Li}}_{x}$F$e_{2}O_{4}$) at 0.2 ≤ x ≤ 0.6. It is clear that increasing to a 90% RH concentration results in a more viscous solution, which can serve as an effective humidity sensor (see Figure 8a,b). At the same time, we compared the humidity-sensing characteristics of three sensors at 100 kHz with a response time of 10 s, which has proven to be the best frequency from 25% RH–95% RH (see Figure 10a,b). As the result shows, a response time of 10 s with a five-volt voltage has a small amount of recovery time (the humidity difference at the same impedance value), which can effectively reduce the error of humidity sensors. 

The sensing fitness of the resistant response of f = 1 MHz is presented in Table 7 and Figure 10. Our experimental testing explained the humidity-sensing mechanism, as suggested in previous studies [1,4,5,11,13,26,32]. At higher frequencies, there was a change in humidity sensors because adsorbed water molecules cannot be polarised with a rapid change in clinical settings [16,33]. The repeatability test with increasing RH proves the suitability of patients with IAD in clinical settings. Therefore, we validated 100 kHz as the operating frequency in the following testing, related to previous studies [1,4,5,31,34]. With a variation of 20–90% RH, the change in five-volt voltage exhibited a relatively small pore size of 0.1 μm. This means that, if a mattress is subjected to a high humidity change, it can be inconvenient and the sensing material of the humidity sensor is not good for patients with IAD in clinical settings [1,2,4,8,28].

Humidification was performed, with a value of 0.43%/HR% at 30 °C, a grain size of 20 nm, and a frequency between 100 Hz and 100 kHz. It was tested to be 0.5% when compared with the photon crystal fibre sensor of graphene oxide [35]. At low RH, a layer of the sensing pad of water molecules was adsorbed to form hydroxyl and sensing performance. Subsequently, as the humidity sensor increased, a layer of water molecules was adsorbed with double hydroxyl bonding. In bulk liquid, $H_{3}O^{+}$þ released $H^{+}$ to the adjacent water molecules and then cycled down to 0.5% RH, with a response time increase of 40 s and a recovery time of approximately 230 s. The low hysteresis in the sensor response was due to the highly hydrophobic structure of the thick film [36].

Response and recovery times constitute one of the significant features of the humidity-sensing mattress. The response time was tested by quickly moving the humidity sensor in a relatively dry environment (35% RH), which achieved 90% of the total impedance change. The sensitivity exhibited a fast response and a recovery time of between 10 s and 30 s, which is the best reported time for a resistive type of humidity-sensing mattress for IAD in clinical settings [24,32,37]. A sputtered metal oxide film (1.2 μm) acted as both an RH and a piezoelectric actuation layer for the electrode. Yang et al. [38], Bian et al. [39] and Chu et al. [40] fabricated humidity sensors using response times of 0.701 nm/RH% and 2.728 nm/RH%, ranging between 30 °C and 110 °C for d = −1 μm. The repeatable sensing mattress is based on humidity, perhaps showing a slight response of 50 nm, but there was a noticeably slower recovery (>600 ms) at 35 °C and a rate of 0.8% HR per minute.

## 5. Conclusions

In conclusion, the design of the humidity-sensing mattress has been tested with regard to the water vapour with RH, AB, PPM, and D/F PT. The testing showed resistance-humidity characteristics of the sensor at 35 °C ($V_{0\;}$ = 30 V, $V_{0\;}$ = 350 mV), the slope at 1.13 V/fF, f = 1 MHz with 20–90% RH, a response time of 20 s at 2 μm, and 300 mm. Repeatable sensitivity demonstrated that the humidity sensor increased to 0.22% RH/°C at 100 °C, and decreased to −0.07% RH/°C at 0.5% RH. The conductivity range was 30% RH with 10 s, a magnitude of 1$0^{7}$–1$0^{4}$ Ω, 1 mol%, and ${\mathrm{CrO}}_{1.5}$ and F$O_{1.5}$, respectively. Further, the feasible humidity sensor integrated into the water vapour was tested by embedding that sensor in the thin-film electrode. The repeatable humidity sensor has many advantages, with a response time of less than 10 s, whilst the addition of 2 mol % of $K^{+}$ can greatly improve the sensitivity over the whole range of RH.

The development of the humidity-sensing mattress is a crucial step in the production of a novel piece of medical technology that provides a faster response, high sensitivity, and recovery time; indeed, it may also be a potential design for practical application. Notably, the design of the humidity-sensing mattress compounded with the thin-film electrode as the RH increased to 90%, showing excellent sensitivity. This property enables the sensor to be applied to medical diagnosis for patients with IAD. When the design has been applied, and the enhanced clinical acceptance of sensing material is a reality, it will be possible to provide and develop a low-cost mattress for IAD in clinical homecare. Once available, this humidity-sensing mattress has broad application prospects in the field of flexible sensors, wearable medical diagnostic devices, health detection, etc.

## Figures and Tables

**Table 1:** Humidity-sensing processing data platform.

Code 1:	Start	Humidity-Sensing Processing Data
1		Preprocessing: warm-up stage → (1 min) to establish the serial communication between ScreenView and the main processor value reading → based on inputs
2		Input: enter → the user data (weight loading, height, age, and gender) in ScreenView
3		Acquire: sensor array values for 1 min with 100 Hz
		Processing: value normalisation
		Calculate: sensor change is sent to ScreenView amongst the contact sensor, historical data, and recorded data
		Output: sensor identification
4	End	

**Table 2:** Volunteer data for the final humidity testing.

No.	Average Humidity Change			Number of ECP			BMI	pH
$P_{1}$	1.3	1.8	1.1	11	23	33	17.6	17.7
$P_{2}$	1.9	2.1	2.3	15	26	28	23.9	17.4
$P_{3}$	2.1	1.9	2.4	18	37	42	27.6	19.1
$P_{4}$	3.5	3.3	3.7	20	36	43	27.2	10.5
$P_{5}$	4.2	3.9	4.1	23	39	44	27.5	12.7
$P_{6}$	1.6	2.4	2.6	16	28	37	24.6	11.1
$P_{7}$	1.8	1.7	1.9	24	27	36	26.1	19.6
$P_{8}$	2.9	2.6	3.2	16	24	33	24.2	18.3
$P_{9}$	3.2	2.7	2.9	19	33	39	27.1	17.4
$P_{10}$	2.7	2.2	2.3	25	40	43	23.7	11.9
$P_{11}$	4.1	3.6	2.6	16	31	37	21.5	12.8
$P_{12}$	3.0	3.3	3.9	13	23	32	20.9	13.6
$P_{13}$	2.9	3.1	3.6	15	27	36	23.7	14.0
$P_{14}$	2.9	2.7	2.9	26	34	35	28.8	13.8
$P_{15}$	2.1	2.6	2.8	17	26	34	25.9	10.9
$P_{16}$	1.8	2.2	3.1	19	29	32	22.5	15.2
$P_{17}$	2.4	2.6	2.8	25	39	45	26.2	16.7
$P_{18}$	2.1	1.9	2.5	16	25	37	26.8	14.6
$P_{19}$	1.9	2.3	2.7	21	27	31	23.7	12.4

**Table 3:** Different RH values.

Saturated Solution	15 °C RH (%)	30 °C RH (%)	45 °C RH (%)	*r* (nm)
LiC1	15	11	10	0.49×
C$H_{3}$COOK	25	22	20	0.57×
Mg$C1_{2}$	35	33	30	1.04×
$K_{3}$C$O_{3}$	45	44	40	1.93×
Mg(N$O_{3}{)}_{2}$	55	52	50	2.17×
Cu$C1_{2}$	65	68	60	3.75×
NaC1	75	75	70	5.02×
KC1	85	85	80	9.78×
$K_{2}{\mathrm{SO}}_{4}$	95	95	9	11.96×

**Table 4:** Average humidity sensor.

Volunteers	Criteria	**P_1_**	**P_2_**	**P_3_**	**P_4_**	**P_5_**	**P_6_**	**P_7_**	**P_8_**	**P_9_**	**P_10_**	**P_11_**	**P_12_**	**P_13_**	**P_14_**	**P_15_**	**P_16_**	**P_17_**	**P_18_**	**P_19_**
Head and neck	DS18B29 (°C)	5.5	7	6	7	8	5.5	6.5	7	7.5	6	5.5	7.5	8.5	7	6	7.5	6	5.5	7
	SHT10 (°C)	5	6	5	6	7.5	5	6	6.5	7	5.5	5	7	8	6.5	5.5	7	5.5	5	6.6
	Humidity SHT10 (%)	15	20	16	20	25	40	35	33	26	17	22	24	26	35	39	28	19	23	27
	FSR400 (kg)	2.5	4.5	5.3	6.7	2.9	5.4	3.5	2.9	4.6	3.2	3.7	3.1	2.8	4.6	5.6	7.6	3.6	3.9	5.3
Shoulders	DS18B20 (°C)	6.5	5.5	7.5	4.5	7.5	3.5	4.5	5.5	6.5	4.5	5.5	7.5	4.5	3.5	6.5	4.5	5.5	6.5	3.5
	SHT_10 (°C)	6	5	6.5	4	6	7.5	4	5	7	4.5	6	5	5.5	4.5	6	7	4.5	6	4.5
	Humidity SHT 10 (%)	7.4	5.5	16	26	33	26	15	12	11	16	19	15	12	11	24	26	17	9.5	14
	FSR400 (kg)	5.6	13.5	10.5	12.4	16.5	12.4	16.7	20.5	16.5	20.5	30.4	12.8	19.5	15.5	12.4	16.4	9.9	20.5	18.3
Lumbar spine	DS18B20 (°C)	5.5	5.5	4.5	6	4	6	5.5	7	8	6.5	4.5	6.5	6	7	5	7	5.5	6.5	7
	SHT_10 (°C)	7.5	5.5	6	7	7	5	6	7.5	6.5	6	7	3.5	6.5	7.5	5	6.5	7.5	7	6
	Humidity SHT 10 (%)	9.6	16	25.6	16.9	25	34	16.8	22.4	18	22	27	26	35	18.3	16.4	20.5	28.9	27	30.6
	FSR400 (kg)	3.4	7.5	16.4	22.2	23	16	19	21	23.5	27.1	8.9	15	12	9.9	23.4	16.4	23.5	19.5	24.5

**Table 5:** Values of temperature and humidity sensors.

Sensor	Criteria	$P_{1}$	$P_{2}$	$P_{3}\;$	$P_{4}$	$P_{5}$	$P_{6}$	$P_{7}$	$P_{8}$	$P_{9}$	$P_{10}$	$P_{11}$	$P_{12}$	$P_{13}$	$P_{14}$	$P_{15}$	$P_{16}$	$P_{17}$	$P_{18}$	$P_{19}$
Head and neck	Temperature DSN (°C)	29.5	29.5	30.5	31.5	29.5	32.5	28.5	29.5	28.5	29.5	30.5	30.5	33.5	30.5	29.5	28.5	29.5	32.5	30.5
	Temperature SHT (°C)	29.5	29.5	30.5	31.5	29.5	31.5	28.5	28.5	28.5	30.5	32.5	30.5	30.5	29.5	30.5	29.5	30.5	33.5	31.5
	Humidity SHT (%)	50	51	52	53	50	53	55	60	54	59	54	49	52	57	62	45	59	58	48
	FSR400 (kg)	2.1	4.2	5.7	6.5	7.3	8.1	9.5	6.1	6.8	6.3	6.6	6.9	7.5	9.5	10.5	11.4	12.6	9.4	7.9
Temperature sensors’ average		**29.9**	**29.8**	**30.7**	**31.2**	**29.6**	**32.4**	**28.7**	**29.4**	**28.8**	**29.7**	**30.3**	**30.9**	**33.6**	**30.9**	**29.9**	**28.3**	**29.8**	**32.5**	**30.9**
Shoulders	Temperature DS (°C)	29.6	29.4	30.8	31.3	29.3	32.8	28.1	29.3	28.8	29.1	30.3	30.6	33.4	30.6	29.4	28.2	29.4	32.9	30.6
	Temperature SHT (°C)	29.4	29.4	30.5	31.4	29.7	31.4	28.5	28.3	28.6	30.9	32.2	30.5	30.6	29.8	30.3	29.7	30.3	33.5	31.7
	Humidity SHT (%)	49	51	50	56	50	53	55	62	54	54	53	50	52	56	63	50	57	53	49
	FSR400 (kg)	7.6	13	15	16	10	17.5	20.5	22	16	18	12	26	23	20.6	19.5	23.9	18.9	15.2	23.3
Temperature sensors’ average		**29.8**	**29.4**	**30.8**	**31.2**	**29.6**	**32.5**	**28.9**	**29.5**	**28.5**	**29.8**	**30.5**	**30.6**	**33.7**	**30.6**	**29.9**	**28.4**	**29.9**	**32.8**	**30.9**
Lumbar spine	Temperature DSN (°C)	30.5	29.5	30.5	30.5	30.5	29.5	28.5	30.5	26.5	29.5	25.5	27.5	29.5	31.5	29.5	27.5	27.5	28.5	30.5
	Temperature SHT (°C)	30.5	30.6	30.6	30.7	30.8	29.7	28.3	30.8	26.6	29.7	25.4	27.6	29.7	31.4	29.6	27.6	27.8	28.6	30.9
	Humidity SHT (%)	51	52	55	56	52	55	56	62	55	57	54	55	57	55	66	54	55	56	52
	FSR400 (kg)	2.5	3.6	10.5	13.7	9.6	11.6	15.6	18.4	11.4	9.6	8.5	10.6	10.8	13.9	16.4	20	18	11.9	14.5

**Table 6:** The comparison of threshold RH testing.

Temperature (°C)	RH (%) Water Vapour	Threshold (%)
20	0.05	>4.05
30	0.24	>4.24
40	0.76	>4.76
50	1.38	>7.30
60	4.95	>10.95
70	19.06	>85.06
80	37.54	>90.54
90	69.19	>129.19

**Table 7:** Characteristics of the humidity sensor for repeatability testing.

Material	Principle	Sensor Element	Operative Range		Response Time
			Temperature (°C)	RH (%)	
Electrolyte	Impedance (ionic)	MgC$r_{2}O_{4}-T_{1}O_{2}$	0–50	1–100	10 s
		$T_{1}O_{2}-V_{2}O_{5}$	0–150	15–100	10 s
		${\mathrm{ZnCr}}_{2}O_{4}-L_{1}\mathrm{Zn}VO_{4}$	0–150	30–90	3 min
	Impedance (electronic)	${\mathrm{Sr}}_{1-x}{\mathrm{La}}_{x}$Sn$O_{3}$	300–500	1–10	2 min
		${\mathrm{Zr}}_{1-}\mathrm{MgO}$	400–700	1–10	10 s
		$A1_{2}O_{3}$	−10–40	1–100	10 s
		${\mathrm{Ta}}_{2}O_{5}-{\mathrm{MnO}}_{2}$	−10–55	1–100	1 min

**Table 8:** Characteristics of humidity sensor repeatability testing.

Material	Sensor Device	μm	μm (av.)	Porosity	Impedance (Ω)		**°** **C**
					**10% RH**	**90% RH**	
$\mathrm{MgC}r_{2}O_{4}-T_{1}O_{2}$	Rectangular water	–	04	35	10 M	10 k	25
	4 × 5 × 025 mm						
$T_{1}O_{2}-V_{2}O_{5}$	Rectangular water	03–10	08	45	13 M	40 k	25
	50 × 28 × 15 mm						
$\mathrm{MgA}I_{5}O_{4}$	Circular water	01–10	05	49	25 M	25 k	25
	10 ∅ × 05 mm						
$\mathrm{MgO}-K_{2}O-$ $Fe_{2}O_{4}$	Circular water	01–04	03	44	1 M	15 k	30
	10 ∅ × 10 mm						
$S_{1}O_{2}$ $+Nn_{2}{\mathrm{CO}}_{3}$	Thick film (20 μm)	1–10	–	–	5 M	5 k	35
	≈10 × 10 mm						
${\mathrm{ZnCr}}_{2}O_{4}-{\mathrm{LiZnVO}}_{4}$	Circular water	01–05	02	15	300 k	5 k	35
	8 ∅ × 02 mm						

## Data Availability

MDPI Research Data Policies.
